# Prevalence of Prostatitis-Like Symptoms and Outcomes of NIH-CPSI in Outpatients with Lifelong and Acquired PE: Based on a Large Cross-Sectional Study in China

**DOI:** 10.1155/2017/3473796

**Published:** 2017-08-07

**Authors:** Daofang Zhu, Xianming Dou, Liang Tang, Dongdong Tang, Guiyi Liao, Weihua Fang, Xiansheng Zhang

**Affiliations:** Department of Urology, The First Affiliated Hospital of Anhui Medical University, Hefei City, Anhui Province, China

## Abstract

Premature ejaculation (PE) is one of the most common sexual dysfunctions, which were associated with prostatitis-like symptoms (PLS). We intended to explore the prevalence of prostatitis-like symptoms and outcomes of National Institutes of Health-Chronic Prostatitis Symptom Index (NIH-CPSI) scores in outpatients with lifelong (LPE) and acquired premature ejaculation (APE). From December 2013 to December 2015, a total of 498 consecutive heterosexual men with PE and 322 male healthy subjects without PE were enrolled. Each of them completed a detailed questionnaire on demographics information, sexual and medical histories, and the NIH-CPSI. Assessment of NIH-CPSI and definition of PLS and PE were used to measure the PLS and NIH-CPSI scores and ejaculatory function for all subjects. Finally, a total of 820 subjects (including 498 men in PE group and 322 men in control group) were enrolled in our study. The mean ages were significantly different between PE and no PE groups. Men with PE reported worse PLS and higher NIH-CPSI scores (*P* < 0.001 for all). Similar findings were also observed between men with LPE and APE. Men with APE also reported higher rates of PLS and scores of NIH-CPSI (*P* < 0.001 for all). Multivariate analysis showed that PLS and NIH-CPSI scores were significantly associated with PE.

## 1. Introduction

Premature ejaculation (PE) is one of the most common sexual dysfunctions, which affects about 20%~30% of male population due to its different definitions [1–4]. Recently, the International Society for Sexual Medicine (ISSM) provided a new definition of PE (lifelong PE [LPE] and acquired PE [APE]) [5]. They included three main characteristics: (1) intravaginal ejaculatory latency time (IELT) always or nearly always about or less than a minute from the first sexual experience (LPE) or a clinically significant and bothersome reduction of IELT, often about or less than 3 minutes (APE); (2) lack of ability to delay ejaculation on all or nearly all vaginal penetrations; and (3) negative personal consequences, such as distress, bothering, frustration, and/or the avoidance of sexual intimacy.

In addition, previous studies have shown that chronic prostatitis (CP) might be contributed to PE. Screponi et al. found that the percentage of prostatic inflammation and chronic bacterial prostatitis in patients with PE was 56.5% and 47.8%, respectively, and there were significant differences between PE patients and controls [6]. The other large observational survey in China showed that PE (especially APE patients) reported worse National Institutes of Health-Chronic Prostatitis Symptom Index (NIH-CPSI) scores and lower IELT than men without PE complaints [1]. Similarly, Liang et al. found that the PE group reported worse NIH-CPSI scores, and the percentage of PE was 64.1% and 36.9% in the prostatitis-like symptom (PLS) and CP group, respectively [7].

Although the associations between CP and PE have been widely studied, they were still not clear. In addition, based on the new definition of LPE and APE, studies on the issues in China were rare. Hence, our study intended to explore the prevalence of PLS and outcomes of NIH-CPSI scores in outpatients with LPE and APE (Table 2).

## 2. Subjects and Methods

An observational and cross-sectional field survey was conducted in the urological department of the Affiliated Hospital of Anhui Medical University. From December 2013 to December 2015, a total of 498 consecutive heterosexual men with PE (including 142 lifelong PE and 356 acquired PE) were recruited from the andrology outpatient's clinic. In addition, 322 male healthy volunteers without PE were enrolled from the health examination center.

To be included in the study, all subjects had to meet the following criteria: (1) age ≥18 years, (2) being in a heterosexual, stable relationship for at least 6 months, and (3) being able to read and speak Chinese. Their sexual and medical history was carefully evaluated by an experienced clinician. Men on medication that could have affected their ejaculation were excluded.

Patients were diagnosed as having LPE and APE according to evidence-based definitions recommended by ISSM. Since several subjective and sensitive personal questions were included in this study, a presurvey was given to a small sample (*N* = 30) to modify the questionnaire and make it comprehensive and easily understood. Before the survey, all subjects were informed about the study and those who participated were asked to provide written consent. Then they completed a questionnaire, including demographic information (e.g., age, education, and occupation), sexual and medical histories (e.g., self-estimated IELT), and the Chinese version of NIH-CPSI [8]. This survey was reviewed and approved by the Anhui Medical University Research Subject Review Board.

Assessment of NIH-CPSI is a reliable, convenient, self-administered index that is widely used across scientific research and clinical studies (including pain symptoms [total of items 1–4], urinary symptoms [total of items 5 and 6], and Quality of life [QOL] impact [total of items 7–9]) [9]. The Chinese version of NIH-CPSI was widely used in the previous studies in China [7, 8, 10–12]. Based on the total of items 1–9, the severity of CPPS was classified as mild (10–14 points), moderate (15–29 points), or severe (≥31 points). In addition, according to Nickel et al. [9], perineal and/or ejaculatory pain/discomfort and a total pain score of NIH-CPSI ≥4 were used to identify men with PLS. Mild symptoms were defined by pain scores of 4 to 7 and moderate or severe symptoms by scores of 8 or greater.

All data were analyzed using the SPSS software version 13.0 (SPSS Inc., Chicago, IL, USA). Descriptive statistics were used to summarize the subjects' characteristics. Data were expressed as mean ± standard deviation or number (percentage) when appropriate. Chi-squared test was used to compare categorical data. The independent *t*-test was used to compare numerical data. The analysis of covariance was used to assess the factors associated with PLS. In addition, the multivariate analysis (adjusted by age, BMI scores, smoking, and exercise) was performed to assess the association between PLS, NIH-CPSI scores, and PE. Odds Ratio (OR) and Confidence Interval (CI) were used to evaluate their associated strength. For all tests, *P* < 0.05 was considered statistically significant.

## 3. Results

A total of 820 subjects (including 498 men in PE group and 322 men in control group) were enrolled in our study. The mean ages in PE (LPE, 28.51%; APE, 71.49%) and no PE groups were 43.41 ± 9.45 and 32.72 ± 8.86 years. In addition, the mean self-estimated IELT in PE groups was 1.49 ± 0.56 minutes, whereas that in control group was 3.16 ± 1.49 minutes. Moreover, the mean self-estimated IELT in men with LPE and APE were 0.84 ± 0.25 minutes and 1.75 ± 0.76 minutes, respectively. Differences between PE and no PE groups were observed with regard to age, body mass index (BMI), self-estimated IELT, and rates of smoking and exercise (*P* < 0.001 for all). Similarly, the differences were also presented between LPE and APE groups. Detailed demographic characteristics of men with and without PE were summarized in Table 1.

Also, significant difference was found between PE and no PE groups, when we analyzed the severity of PLS and prostatitis symptoms and NIH-CPSI scores (*P* < 0.001 for all). The incidence of PLS in men with PE was 36.54%, whereas that in no PE group was 13.36%. The mean NIH-CPSI scores in LPE and APE were 24.47 ± 8.44 and 33.26 ± 9.21, respectively. That is to say, men with PE reported worse PLS and higher NIH-CPSI scores. In addition, similar findings were also observed between men with LPE and APE. Men with APE also reported higher rates of PLS and scores of NIH-CPSI (including total and subdomain scores) (*P* < 0.001 for all).

Finally, with the analysis of covariance, significant associations were found between PLS and some factors (including age, BMI scores, smoking, exercise, and NIH-CPSI scores) (*P* < 0.001 for all). However, the multiple logistic regression (adjusted by age, BMI scores, smoking, and exercise) showed the association between PLS and NIH-CPSI scores and PE (PLS [OR = 2.75, CI = 2.26–4.33] and NIH-CPSI scores [**15–30**: OR = 1.72, CI = 1.43–2.65;** 31–43**: OR = 2.83, CI = 2.45–4.52]) (Table 3).

## 4. Discussion

Based on the new definition of PE, this is the first study to investigate the incidence of PLS and outcomes of NIH-CPSI in outpatients with LPE and APE. In addition, the new PE classification provides a better perspective of the epidemiology, pathophysiology, etiology, and treatment of various forms of PE. In our study, we found that the prevalence of PLS and NIH-CPSI scores in PE group were higher than those in no PE group. In addition, patients with APE reported higher incidence of PLS and NIH-CPSI scores than men with LPE.

PE was known to be a multifactorial sexual dysfunction [10, 13–15]. Previous studies have showed that it might be associated with prostatitis symptoms. After evaluating a consecutive series of 244 men with couple infertility, Lotti et al. [16] found that PE (evaluated by PE diagnostic tool [PEDT]) was positively associated with prostatitis symptoms. PEDT score was related to the total and subdomain scores of NIH-CPSI. In an observational study of 1,5000 Chinese men, Liang et al. [7] found that 64.07% of patients with prostatitis-like symptoms reported PE, significantly more than one might expect in a population ranging from 51 to 60 years. In addition, participants with PE versus men without PE reported worse NIH-CPSI total (37.2 ± 4.6 versus 18.2 ± 5.6) and subdomain scores (including pain symptoms, urinary symptoms, and QOL subscores). Relationships between PE and CP symptoms were obvious in their study. Another study conducted by Bartoletti et al. [17] showed that chronic pelvic pain syndrome (CPPS) had a negative influence on male sexual function. Compared with the control group, the incidence of PE was higher in patients with CPPS. Similar findings were also observed in our survey. Results from our survey showed that men with PE reported higher incidence of PLS than men without PE. In addition, NIH-CPSI scores in PE groups were higher than those in control group. However, the difference in prevalence rates might be explained by the cultural and religious differences between the Chinese and Western patient populations used in the respective studies.

In addition, association between PLS and age, BMI scores, lifestyle, and NIH-CPSI scores was showed in our study. Men with PLS might report older age, higher BMI scores and NIH-CPSI scores, and unhealthy lifestyle (e.g., smoking, no exercise). Because the definition of PLS includes perineal or ejaculatory discomfort and a total pain score of NIH-CPSI ≥4, we speculated that factors associated with perineal or ejaculatory discomfort (e.g., age, smoking) and NIH-CPSI scores might influence the incidence of PLS. However, previous studies on the issue were few in China, and further studies were needed.

We also investigated the incidence of PLS and outcomes of NIH-CPSI in outpatients with LPE and APE, respectively. Our study showed that APE patients reported higher PLS and NIH-CPSI scores than LPE patients. Similarly, Serefoglu et al. [18] found that the incidence of CP in men with APE were higher than men with LPE. In another study based on 690 middle-aged men complaining of ejaculating prematurely, results reported that the correlation of IPSS with self-estimated IELT of APE patients was stronger than that with LPE patients [19]. Gao et al. [20] also found that negative relationships between total and subdomain scores of NIH-CPSI and IELT were stronger in men with APE than LPE. Hence, our results confirmed the above findings, and further studies were also needed.

There are also several limitations in the present study. Subjects participating in this study completed the questionnaires face to face with investigators, and so many patients may exaggerate or reduce their symptoms due to embarrassment when dealing with this sensitive personal problem. Particularly, the NIH-CPSI may influence the accuracy of our findings. Other methods of obtaining data from these patients, such as Internet-based surveys, should be considered for future studies. And we did not take any laboratory examinations and ultrasonography to exclude other diseases, for example, benign prostatic hyperplasia. A further and more accurate definition of CP is needed to indicate the exact prevalence. Finally, we have chosen the control group from our medical examination center; a community-based investigation should be made in further study for more accurate study.

## 5. Conclusion

Patients with PE had higher incidence of PLS and CP and had higher NIH-CPSI scores than that of the control subjects. Incidence of PLS in the APE group was higher than that of LPE group.

## Figures and Tables

**Table 1:** Demographic characteristics in men with and without PE.

Factors	ALL (*n* = 820)	PE group (*n* = 498)	No PE group (*n* = 322)	*P* ^*∗*^ value	LPE (*n* = 142)	APE (*n* = 356)	*P* ^*∗∗*^ value
*Age, years*	39.21 ± 9.25	43.41 ± 9.45	32.72 ± 8.86	**<0.001**	38.72 ± 9.17	45.28 ± 10.83	**<0.001**
*BMI, scores*	25.06 ± 3.14	26.59 ± 3.47	22.69 ± 2.75	**<0.001**	23.74 ± 3.82	27.73 ± 3.65	**<0.001**
*IELT, minutes*	2.15 ± 1.28	1.49 ± 0.56	3.16 ± 1.49	**<0.001**	0.84 ± 0.25	1.75 ± 0.76	**<0.001**
*Smoking*	487 (59.39%)	336 (64.47%)	151 (46.89%)	**<0.001**	76 (53.52%)	260 (73.03%)	**<0.001**
*Exercise*	347 (42.32%)	184 (36.95%)	163 (50.62%)	**<0.001**	66 (46.48%)	118 (33.15%)	**<0.001**
*Educational status*				**0.612**			**0.994**
Others	117 (14.27%)	67 (13.45%)	50 (15.53%)		20 (14.08%)	47 (13.20%)	
Primary education	171 (20.85%)	100 (20.08%)	71 (22.05%)		28 (19.72%)	72 (20.22%)	
High school	330 (40.24%)	202 (40.56%)	128 (39.75%)		57 (40.14%)	145 (40.73%)	
Higher education	202 (24.63%)	129 (25.90%)	73 (22.67%)		37 (26.06%)	92 (25.84%)	
*Occupational status*				**0.463**			**0.880**
Student	192 (23.41%)	110 (22.09%)	82 (25.47%)		32 (22.54%)	78 (21.91%)	
Unemployed	198 (24.15%)	121 (24.30%)	77 (23.91%)		31 (21.83%)	90 (25.28%)	
Employed	333 (40.61%)	202 (40.56%)	131 (40.68%)		60 (42.25%)	142 (39.89%)	
Retired	97 (11.83%)	65 (13.05%)	32 (9.94%)		19 (13.38%)	46 (12.92%)	

PE = premature ejaculation; BMI = body mass index; IELT = intravaginal ejaculatory latency time; LPE = lifelong PE; APE = acquired PE. ^*∗*^Differences between PE complaint and no PE complaint were assessed by Chi-square test or *t*-test, as appropriate. ^*∗∗*^Differences between LPE and APE were assessed by Chi-square test or *t*-test, as appropriate.

**Table 2:** Outcomes of PLS and NIH-CPSI in men with LPE/APE.

	ALL (*n* = 820)	PE group (*n* = 498)	No PE group (*n* = 322)	*P* ^*∗*^ value	LPE (*n* = 142)	APE (*n* = 356)	*P* ^*∗∗*^ value
*PLS*				*<0.001*			*<0.001*
Mild (4–7)	134 (16.34%)	102 (20.48%)	32 (9.94%)		23 (16.20%)	79 (22.19%)	
Moderate or severe (≥8)	91 (11.10%)	80 (16.06%)	11 (3.42%)		18 (12.68%)	62 (17.42%)	
*NIH-CPSI, scores*							
Total scores	19.74 ± 6.85	30.75 ± 8.89	2.72 ± 1.08	*<0.001*	24.47 ± 8.44	33.26 ± 9.21	*<0.001*
Pain symptoms	10.44 ± 4.32	16.44 ± 5.64	1.15 ± 0.41	*<0.001*	14.46 ± 5.04	17.23 ± 6.12	*<0.001*
Urinary symptoms	4.92 ± 1.88	7.43 ± 3.06	1.03 ± 0.40	*<0.001*	5.27 ± 2.85	8.29 ± 3.33	*<0.001*
Quality of life impact	4.37 ± 1.74	6.85 ± 2.16	0.54 ± 0.16	*<0.001*	4.74 ± 1.45	7.74 ± 2.36	*<0.001*
*NIH-CPSI, scores*				*<0.001*			*<0.001*
Mild (0–14)	622 (75.85%)	314 (63.05%)	308 (95.65%)		98 (69.01%)	216 (60.67%)	
Moderate (15–30)	142 (17.32%)	128 (25.70%)	14 (4.35%)		32 (22.54%)	96 (26.97%)	
Severe (31–43)	56 (6.83%)	56 (11.24%)	0 (0.00%)		12 (8.45%)	44 (12.36%)	

PE = premature ejaculation; LPE = lifelong PE; APE = acquired PE; PLS = prostatitis-like symptoms; NIH-CPSI = National Institute of Health-Chronic Prostatitis Symptoms Index. ^*∗*^Differences between PE and no PE groups were assessed by *t*-test. ^*∗∗*^Differences between LPE and APE were assessed by *t*-test.

**Table 3:** Association between PLS, NIH-CPSI scores, and PE.

Factors	*P* value	OR^*∗*^	95% CI	
			Lower	Upper
*PLS*				
*No*	*1*			
*Yes*	*<0.001*	**2.75**	2.26	4.33
*NIH-CPSI, scores*				
*0–14*	*<0.001*	**1**		
*15–30*	*<0.001*	**1.72**	1.43	2.65
*31–43*	*<0.001*	**2.83**	2.45	4.52

PE = premature ejaculation; PLS = prostatitis-like symptoms; NIH-CPSI = National Institute of Health-Chronic Prostatitis Symptoms Index; OR = Odds Ratio; CI = Confidence Interval. ^**∗**^Date were assessed by the multiple logistic regression, when adjusted by age, BMI scores, smoking, and exercise.
